# Poxin-deficient poxviruses are sensed by cGAS prior to genome replication

**DOI:** 10.1099/jgv.0.002036

**Published:** 2024-10-21

**Authors:** Sian Lant, Alasdair J. M. Hood, Joe A. Holley, Ailish Ellis, Lucy Eke, Rebecca P. Sumner, David O. Ulaeto, Carlos Maluquer de Motes

**Affiliations:** 1 Department of Microbial Sciences, University of Surrey, Guildford, GU2 7XH, UK; 2 CBR Division, Defence Science and Technology Laboratory, Salisbury, SP4 0JQ, UK

**Keywords:** cGAS, *Ectromelia* virus, genome uncoating, IRF3, orthopoxviruses, STING, vaccinia virus

## Abstract

Poxviruses are dsDNA viruses infecting a wide range of cell types, where they need to contend with multiple host antiviral pathways, including DNA and RNA sensing. Accordingly, poxviruses encode a variety of immune antagonists, most of which are expressed early during infection from within virus cores before uncoating and genome release take place. Amongst these antagonists, the poxvirus immune nuclease (poxin) counteracts the cyclic 2′3′-GMP-AMP (2′3′-cGAMP) synthase (cGAS)/stimulator of interferon genes DNA sensing pathway by degrading the immunomodulatory cyclic dinucleotide 2′3′-cGAMP, the product of activated cGAS. Here, we use poxviruses engineered to lack poxin to investigate how virus infection triggers the activation of STING and its downstream transcription factor interferon-responsive factor 3 (IRF3). Our results demonstrate that poxin-deficient vaccinia virus (VACV) and ectromelia virus (ECTV) induce IRF3 activation in primary fibroblasts and differentiated macrophages, although to a lower extent in VACV compared to ECTV. In fibroblasts, IRF3 activation was detectable at 10 h post-infection (hpi) and was abolished by the DNA replication inhibitor cytosine arabinoside (AraC), indicating that the sensing was mediated by replicated genomes. In macrophages, IRF3 activation was detectable at 4 hpi, and this was not affected by AraC, suggesting that the sensing in this cell type was induced by genomes released from incoming virions. In agreement with this, macrophages expressing short hairpin RNA (shRNA) against the virus uncoating factor D5 showed reduced IRF3 activation upon infection. Collectively, our data show that the viral genome is sensed by cGAS prior to and during genome replication, but immune activation downstream of it is effectively suppressed by poxin. Our data also support the model where virus uncoating acts as an immune evasion strategy to simultaneously cloak the viral genome and allow the expression of early immune antagonists.

## Introduction

Nucleic acids are potent inducers of innate immunity and are recognized by a set of germ-line-encoded receptors generally known as pattern-recognition receptors (PRRs). Engagement of PRR by pathogen-associated molecular patterns (PAMPs) results in inflammatory and antiviral responses, including the production of type I interferon (IFN) and the expression of IFN-stimulated genes. Multiple PRRs have been described with reported abilities to sense RNA or DNA in different cellular compartments and impact disease pathology [1,4]. Recognition of RNA in the cell cytosol is mediated by retinoic acid-inducible gene-I (RIG-I) and melanoma differentiation-associated gene-5 (MDA-5), both of which associate with the mitochondrial antiviral signalling (MAVS) to mediate the activation of IFN-responsive factors (IRFs) and NF-κB-light-chain-enhancer of activated B cells [3]. Recognition of cytosolic DNA is largely mediated by the cyclic 2′3′-GMP-AMP (2′3′-cGAMP) synthase (cGAS). Activated cGAS generates 2′3′-cGAMP, which potently induces IRF3 and NF-κB activation via the stimulator of IFN genes (STING) and the TANK-binding kinase-1 (TBK-1) [1]. These transcription factors are therefore pivotal for the induction of IFN antiviral responses upon RNA and DNA, but how they become activated during infection with viruses is often confounded by viral antagonism.

Poxviruses are a family of viruses infecting a large variety of species. The genus orthopoxvirus (OPXV) encompasses the prototypic member of the family, vaccinia virus (VACV), as well as a number of rodent viruses, including cowpox virus (CPXV), *Ectromelia* virus (ECTV) and the recently resurged monkeypox virus (MPXV) [5,6]. OPXV harbours large dsDNA genomes of about 200 kbp that are exclusively replicated in the cell cytosol. This unique feature makes OPXV particularly susceptible to host cytosolic DNA sensors, which are counteracted by these viruses through several mechanisms [7,8]. An immune evasion strategy used by OPXV is the expression of an enzyme capable of degrading 2′3′-cGAMP termed poxvirus immune nuclease or poxin [9,10]. Poxin is a 24.6 kDa protein acid encoded by the gene *B2L* in the VACV nomenclature. In most OPXV, including ECTV, CPXV and MPXV, poxin is expressed as a 57.5 kDa protein due to its C-terminal fusion with a second protein domain similar to the mammalian Schlafen proteins, which is historically known as viral Schlafen (vSlfn) [11]. Previous work from us and others has shown that poxin contributes to virulence in VACV [9] and ECTV [12], where a remarkable 5-log reduction in lethal dose was observed for a virus lacking the vSlfn gene (ECTV.ΔvSlfn).

In a mouse model, infection with ECTV.ΔvSlfn triggered a robust IFN response, resulting in a drastic attenuation relative to parental WT ECTV, and this difference was abolished in mice unable to respond to IFN [12]. Furthermore, the attenuation of ECTV.ΔvSlfn *in vivo* correlated with the inability of this virus to dampen the IRF3 activation in response to cytosolic DNA sensing stimulation [12]. These data supported the idea that the cGAS/STING pathway chiefly controls OPXV immune sensing and that the cGAS activation and production of 2′3′-cGAMP are essential for protection against lethal infection. We also observed that ECTV.ΔvSlfn infection was sufficient to result in detectable activation of STING and IRF3, and this was abolished in cells deficient for cGAS or STING [12]. These observations indicate that poxin is a critical viral antagonist to prevent innate immune sensing, and even in the presence of other viral immunomodulators, the absence of poxin results in a measurable activation of the cGAS/STING DNA sensing pathway.

Like most viruses, poxviruses require the release of the viral genome from the incoming particle prior to its replication and encapsidation into new virions [13,15]. Poxvirus particle structure is unique in that it carries two proteinaceous lateral bodies between the viral membrane and the core wall. Upon entry and disassembly of the lateral bodies, poxviruses follow a complex process that mediates the release of the viral genome from the proteinaceous wall. This process, known as genome uncoating, requires viral early gene transcription and protein synthesis [16]. Early transcription occurs within the virion cores, which are able to import ribonucleotides and export capped and polyadenylated messenger RNA [17,20]. One of these early viral proteins, named D5, is essential to mediate genome release and the formation of DNA pre-replication foci [21]. Besides D5, at least three viral proteins with redundant functions are also involved in virus uncoating and DNA replication [22]. In addition, this process requires a functional cellular proteasome to mediate the degradation and rupture of the core wall and liberation of the genome [23,26]. Dissolution of the core eventually deposits the viral genome into discrete cytoplasmic structures known as viral or replication factories [16,27]. As replication progresses, these factories expand in size and become surrounded by endoplasmic reticulum (ER) membranes [28,30].

In this study, we reasoned that poxin-deficient viruses could be useful tools to understand how OPXV infections are sensed and the origin of the DNA PAMPs recognized by cGAS during infection. Using VACV and ECTV engineered to lack *B2L* or vSlfn, we show that while replicated genomes induce immune activation in fibroblasts, macrophages are able to sense genomes released from incoming virions before genome replication takes place. These results indicate that the complex process of virus uncoating and genome release that occurs after OPXV entry is critical to counteract cellular DNA sensing activation via the expression of early immune antagonists, including poxin, and that antiviral strategies aimed at disrupting this process would have the added benefit of activating a protective immune response.

## Methods

### Cells and viruses

Human foetal foreskin fibroblasts (HFFF), BS-C-40 and BHK-21 cells were grown in Dulbecco’s Modified Eagle Medium (DMEM, Life Technologies) supplemented with 10% heat-inactivated FCS (Biowest), 100 U ml^−1^ penicillin and 100 µg ml^−1^ streptomycin (Pen/Strep, Life Technologies). THP-1 cells expressing *Gaussia* luciferase (GLuc) under the control of the promoter of the IFN-induced protein with tetratricopeptide 1 (IFIT1) have been described [31] and were grown in Roswell Park Memorial Institute (RPMI) 1640 (Life Technologies) supplemented with 15% FCS and Pen/Strep. THP-1 cells knocked out for STING or MAVS have been previously described [32,33]. THP-1 cells knocked out for cGAS were a gift from Prof. Greg Towers (University College London, UCL). The following viruses were used: VACV strain Western Reserve (WR), VACV strain Copenhagen (COP), modified vaccinia Ankara (MVA), VACV.A5.GFP [34], VACV.F13.V5 [35], ECTV strain Naval and ECTV.ΔvSlfn [12]. MVA was grown and titrated in chicken embryo fibroblasts. All other viruses were expanded and titrated in BS-C-40. All viruses were purified through a 36% sucrose cushion before use [36].

### Reagents

Phorbol 12-myristate 13-acetate (PMA, Santa Cruz Biotechnology) was dissolved in DMSO at 10 mg ml^−1^. Herring Testes DNA (HT-DNA, Sigma) was dissolved in water at 2 mg ml^−1^. The 2′3′-cGAMP (InvivoGen) was dissolved in water at 1 mg ml^−1^. Sendai virus (SeV) was a gift from Steve Goodbourn (St George’s University of London, UK). Lipopolysaccharide (LPS) (InvivoGen) was dissolved in water at 5 mg ml^−1^. Cytosine arabinoside (AraC, Sigma) was dissolved in water at 20 mg ml^−1^. Mycophenolic acid (25 µg ml^−1^), hypoxanthine (14 µg ml^−1^) and xanthine (250 µg ml^−1^) were from Sigma.

### Generation of recombinant VACV lacking *B2R*

Recombinant VACV lacking *B2R* was generated by transient dominant selection as previously described [37,38] with minor modifications. Briefly, 1.75×10^6^ HEK293T cells were seeded into a T25 flask and infected with VACV WR at 0.1 p.f.u. cell^−1^ and subsequently transfected with 5 µg of a pUC13 transfer vector containing a sequence homologous to the *B2R* locus except lacking the coding sequence. The cells were incubated for 48 h at 37 °C and progeny virus harvested by freeze–thawing cells. This mixed pool was used to infect a monolayer of BS-C-40 cells in a six-well plate pretreated with selective media (in the presence of mycophenolic acid, xanthine and hypoxanthine) for 1 h before replacing the inoculum with 2 ml of 1× Minimum Essential Medium (MEM) supplemented with 1% low gelling temperature agarose (Sigma) and selective drugs. After 48 h infection, plaques were visualized using a Zeiss TV100 fluorescence microscope (Zeiss), and fluorescent plaques were picked and taken forward for further infections. After three rounds of plaque purification to ensure the purity of the intermediate virus, plaque purification was carried out in the absence of selective drugs, and, in this case, non-fluorescent plaques were isolated. After further three rounds of isolating non-fluorescent plaques, the resolved viral stocks were validated for the desired recombination event by PCR. BS-C-40 monolayers in a 24-well plate were infected with the resolved virus for 48 h, and subsequently, the cells were removed from the wells by scraping. This cell suspension was treated with proteinase K (Qiagen) at 65 °C for 15 min and then inactivated by incubating at 85 °C for 10 min. This was then used as a template for PCR using GoTaq (Promega) in a Veriti Thermal Cycler (Applied Biosystems), and products were run on a 1% agarose (Sigma) gel and separated by electrophoresis.

### Virus growth curves and plaque size

For the multi-step growth curve, confluent BS-C-40 monolayers in 24-well plates were infected with VACV WR or VACV.ΔB2 at 0.1 p.f.u. cell^−1^ in triplicate on ice for 1 h to allow viral particles to adhere to cells. After this, the plates were returned to the 37 °C incubator, and the virus was harvested at 0, 12, 24 and 48 h post-infection (hpi) by scraping the cells from the wells in their media. Virions were released from the cells by freeze–thawing thrice and sonicating each sample. These samples were assessed for viral titre by plaque assay on BS-C-40 monolayers. The plaque size of each virus was assessed by taking stained plaque assays performed in parallel and scanning them using an Epson Perfection V700 (Epson) photo scanner. These images were converted into binary images using ImageJ software (National Institutes of Health). The scale was set using the diameter of the six-well plate, which is 35 mm, and the size of the plaques measured automatically. Data were manually validated to remove non-plaque disruptions of the monolayer, and 20 plaques were chosen at random to quantify.

### Production of serum against B2

A codon-optimized sequence for the VACV *B2R* gene was obtained from GeneArt (Invitrogen) and cloned into pTZ_E12 (Trenzyme GmbH) downstream of the 9× histidine tag and recognition sequence for the tobacco etch virus. His-tagged B2 was expressed in *Escherichia coli* Rosetta 2 (DE3) cells upon the addition of IPTG at 23 °C for 16 h. Bacterial cell pellets were resuspended in lysis buffer (50 mM Tris/HCl pH 7.5, 100 mM NaCl and 10 mM imidazole) supplemented with protease inhibitors (Roche) and DNAse I (PanReac). Upon sonication, lysates were centrifuged (25 000 ***g***, 20 min and 4 °C), and the soluble fraction was purified through a nickel-NTA chromatography column (GE Healthcare). After washing with lysis buffer, the bound protein was eluted in lysis buffer with an increasing gradient of imidazole concentrations and analysed by silver staining and anti-histidine immunoblotting as previously reported [39]. Elution fractions were pooled and subjected to buffer exchange to 50 mM Tris/HCl, 50 mM NaCl and pH 7.5 using HiPrep desalting columns (Cytiva). Purified protein was used to inoculate rabbits, and polyclonal serum was collected on different days after immunization (Invitrogen). Positive serum samples were pooled and used to immunoblot lysates infected with VACV or VACV.ΔB2 at dilution 1 : 2000.

### Reporter activity assays

Luciferase assays using THP-1-IFIT1-Gluc cells were performed as previously described [40]. Briefly, cells were seeded in 96-well plates at a density of 5×10^4^ cells per well in the presence of 50 ng ml^−1^ of PMA. After 48 h, the cells were infected and/or stimulated by agonist transfection with Lipofectamine 2000 (Invitrogen), as indicated in the figure legends. After 16 h, the luciferase activity was measured in a Clariostar plate reader (BMG Biotech) in the presence of 2 µg ml^−1^ coelenterazine (NanoLight Technology). Data were normalized to mock-infected samples and presented as a fold increase. Statistical significance was determined using an unpaired Student’s t-test with Welch’s correction where appropriate using GraphPad Prism statistical software.

### Generation of D5 knock-down cell line

The small interfering RNA (siRNA) sequence targeting the VACV *D5L* gene (originally described by Kilcher *et al.* [21]) was transformed into short hairpin RNA (shRNA) and was cloned into the lentivector HIV.SIREN [41]. A non-targeting control shRNA was also used [42]. For lentivirus production, 10 cm dishes of HEK293T were transfected with 1 µg of p8.91 packaging plasmid [43], 1 µg of the vesicular stomatitis virus G glycoprotein expression plasmid pMDG (GenScript) and 1.5 µg of SIREN plasmid using FuGENE 6 transfection reagent (Promega) according to the manufacturer’s instructions. This mixture was incubated at room temperature for 20 min. Then, the transfection mixture was added to the cultured cells dropwise while gently swirling. After 24 h, the media was removed and replaced with 8 ml fresh DMEM containing 2% FCS. Virus supernatants were collected at 48 and 72 h post-transfection and subsequently pooled, filtered using a 0.45 µm filter (Merck) and stored at −80 °C. For transduction, 5×10^5^ THP-1 cells were incubated with 1 ml of lentivirus medium supplemented with 8 µg ml^−1^ polybrene and centrifuged at 1000 ***g*** for 1 h. Cells were then seeded into six-well plates with complete RPMI media and incubated at 37 °C for a further 48 h. After this period, end-point dilutions were performed in 96-well plates containing medium supplemented with 1 µg ml^−1^ puromycin. Clonal cell lines were subsequently expanded and functionally screened by microscopy using VACV.A5.GFP. Selected cell lines were further expanded and validated by quantitative reverse-transcription PCR and immunoblotting.

### Quantitative reverse-transcription PCR

Cells were collected and washed with ice-cold PBS. Total RNA was isolated using the RNeasy Mini Kit (Qiagen), including a DNAse I step (Qiagen), following the manufacturer’s instructions. RNA concentration was determined by NanoDrop (ThermoFisher), and 1 µg of total RNA was used to generate cDNA using SuperScript III Reverse Transcriptase (Invitrogen), according to the manufacturer’s protocol. cDNA was diluted 1 : 5 in water and used as a template for real-time PCR using SYBR Green PCR Master Mix in a QuantStudio 5 (Applied Biosystems). The expression of *D5L* was normalized to *RNA18S1* and presented as a percentage reduction over the control. Primers 5′-GGACCTGGTAGACGGAATGT-3′ (forward) and 5′-ACATCCTTTGGTTAGCACCGC-3′ (reverse) were used for *D5L* detection, whereas primers 5′-GTAACCCGTTGAACCCCA-3′ (forward) and 5′-CCATCCAATCGGTAGTAGG-3′ (reverse) were used for *RNA18S1*.

### Quantitation of viral DNA

The total DNA from infected cells was isolated using the QIAamp DNA Blood Mini Kit, including proteinase K and RNAse steps, as indicated in the manufacturer’s instructions. Purified DNA concentration was determined by NanoDrop (ThermoFisher) and used as a template for real-time qPCR using SYBR Green PCR Master Mix in a QuantStudio 5 instrument (Applied Biosystems). Viral genomic DNA was measured with primers targeting the *F13L* (forward 5′-CGATTTACTGTGGCTAGATAC-3′ and reverse 5′-ATATCACTTCGGCAAATTTCG-3′) and the *A26L* loci (forward 5′-ATCTCCCATGTGGTGGAATAC-3′ and reverse 5′-GTTGATAGGTTAGAACATCAC-3′) and normalized to cellular genomic DNA, which was detected using primers for the intronless *IFNB1* gene (forward 5′-ACATCCCTGAGGAGATTAAGCA-3′ and reverse 5′-GCCAGGAGGTTCTCAACAATAG-3′). Data were presented as ΔCt.

### Immunoblotting

Cells were lysed in radioimmunoprecipitation assay (RIPA) buffer supplemented with 1 U ml^−1^ Benzonase (Merck) and denatured at 37 °C in the presence of loading buffer. Denatured samples were resolved by SDS-PAGE and transferred to nitrocellulose membranes (GE Healthcare) using a Trans-Blot semi-dry transfer unit (Bio-Rad). Membranes were blocked in PBS supplemented with 0.1% Tween and 5% skimmed milk (Sigma) and subjected to immunoblotting with the following primary antibodies at the indicated dilutions: cGAS (Cell Signaling Technology, 1 : 1000); phosphorylated STING (pSTING) Ser366 (Cell Signaling Technology, 1 : 1000); STING (Cell Signaling Technology, 1 : 1000); IRF3 (Abcam, 1 : 1000); phosphorylated IRF3 (pIRF3) Ser386 (Abcam, 1 : 1000); phosphorylated TBK1 (pTBK1) Ser 172 (Cell Signaling Technology, 1 : 1000); TBK1 (Cell Signaling Technology, 1 : 1000); α-tubulin (Upstate Biotech, 1 : 10 000); β-actin (Protein Tech, 1 : 2000); C6 [44]; D8 [45] and F13 [46]. Primary antibodies were detected using IRDye-conjugated secondary antibodies in an Odyssey infrared imager (LI-COR Biosciences).

## Results

### IRF3 activation in response to MVA is entirely dependent on cGAS and STING

**Fig. 1.**
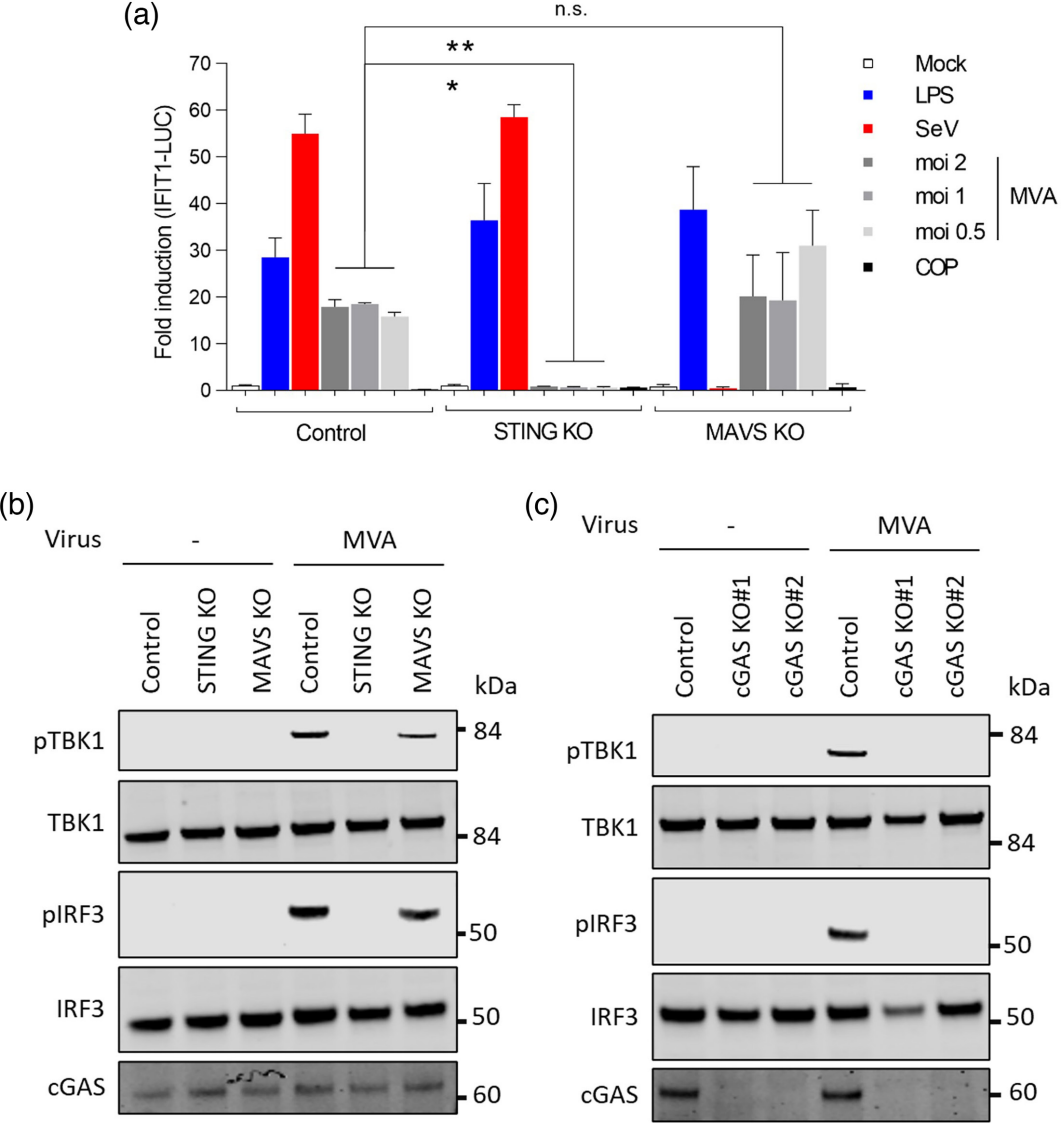
Activation of IRF3 by MVA is entirely dependent on cGAS and STING. (**a**) PMA-differentiated THP-1-IFIT1-GLuc cells edited to knockout (KO) STING or MAVS were infected in triplicate with MVA at the indicated MOI, VACV strain COP at MOI 2 or stimulated with LPS or SeV as controls. Luciferase activity was measured after 16 h and presented as a fold increase over control conditions. Data are representative of at least three experiments and presented as means ± sd. Statistical analyses were performed using Student’s t-test with Welch’s correction where appropriate. (**b, c)** PMA-differentiated THP-1 cells lacking STING or MAVS (**b**) or cGAS (**c**) were infected with MVA (MOI 5). After 10 h, cells were lysed in RIPA buffer and subjected to immunoblotting against the indicated proteins. Data are representative of three experiments with similar results. Molecular weight markers are shown. *P < 0.05, **P < 0.01. n.s., non-significant.

Using differentiated THP-1 cells harbouring an IFN-responsive luciferase reporter (THP-1-IFIT-1-Gluc), we had previously shown that the reporter activation and cytokine production in response to MVA were substantially suppressed when cGAS or STING expression was knocked down by shRNA [40]. To complement this observation, we repeated these experiments in differentiated THP-1 knocked out for STING or MAVS by CRISPR/Cas-9 technology, which has been previously described and validated [32,33]. The absence of MAVS completely abolished the response to SeV, an RNA virus, but had a negligible effect on MVA-induced IFIT1-Gluc activation (Fig. 1a). Conversely, the absence of STING completely abolished MVA-induced responses but had no impact on responses induced by SeV or LPS. The dependence on cGAS and STING, but not MAVS, was then confirmed by immunoblotting of pTBK1 (Ser 172) and pIRF3 (Ser 396), well-established activation markers for the cGAS/STING pathway, in knock-out THP-1 cells (Fig. 1b, c). These results were in agreement with several studies demonstrating that MVA is sensed by cGAS and STING in multiple cell types [47,50].

### VACV and ECTV lacking the viral 2′3′-cGAMP nuclease induce differential IRF3 activation

**Fig. 2.**
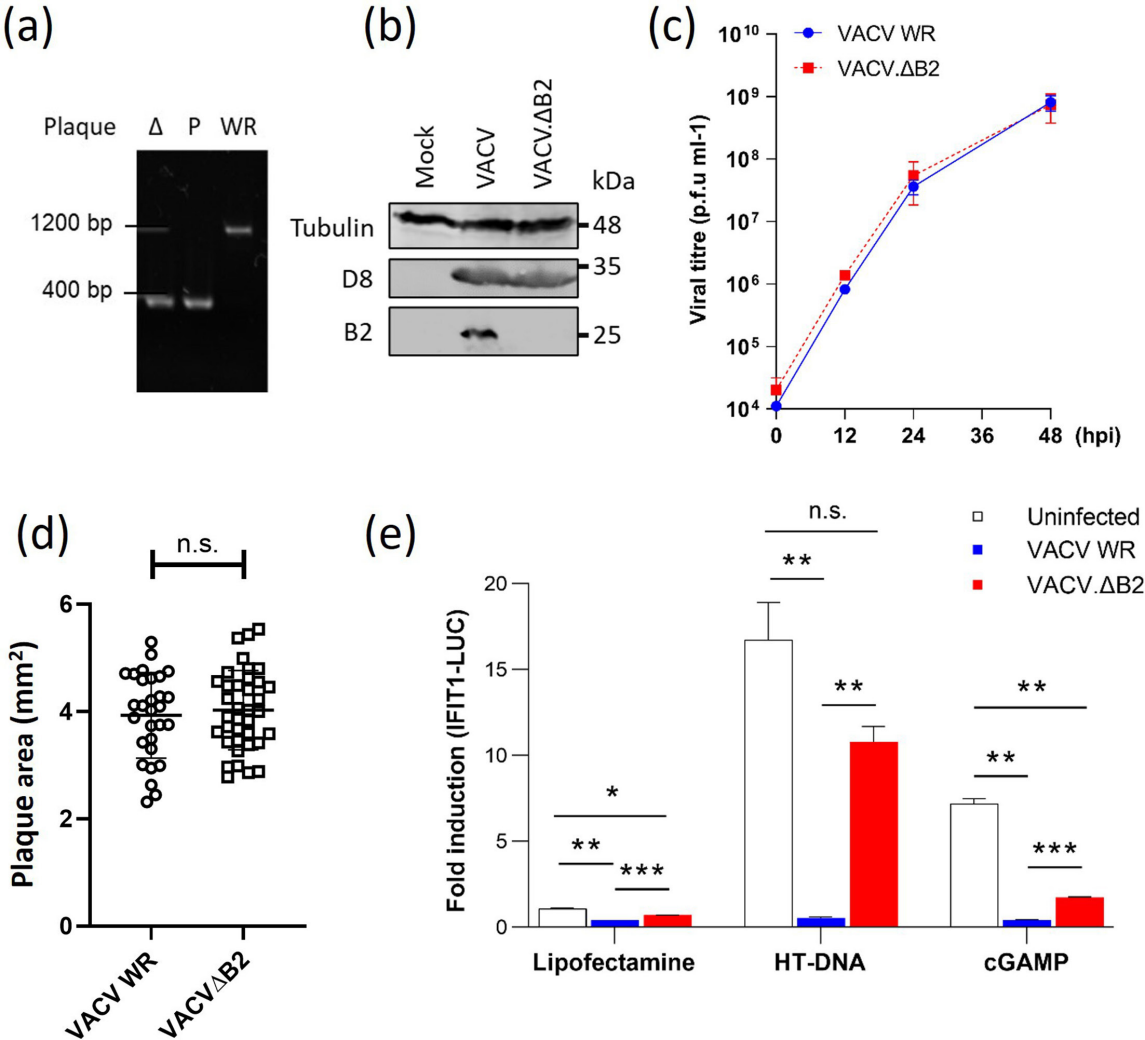
VACV lacking poxin is unable to antagonize DNA sensing. (a) PCR for gene *B2R* in VACV WR, a virus knocked out for B2R (Δ), and the plasmid used to create the knock-out virus by recombination (P). The absence of the gene *B2R* results in a reduced amplicon size. (b) Immunoblotting for B2 in lysates infected with VACV and VACV.ΔB2. Lysates were also probed for α-tubulin and the viral protein D8 as an infection control. Molecular weight markers are shown. (c) Growth kinetics of VACV and VACV. ΔB2 in BS-C-40 cells. Cells were infected with the indicated viruses at an MOI 0.01 and progeny titrated at 12, 24 and 48 hpi. (d) The size of VACV and VACV. ΔB2 plaques in BS-C-40 cells were measured at 48 hpi. (e) PMA-differentiated THP-1 cells were infected with VACV and VACV. ΔB2 at MOI 2. After 6 h, the cells were transfected with 1 µg ml^−1^ HT-DNA or 1 µg ml^−1^ 2′3′-cGAMP and incubated for a further 16 h. Luciferase activity was measured and presented as fold induction over mock-treated uninfected conditions. Data are representative of at least three experiments and presented as means ± sd. Statistical analyses were performed using Student’s t-test with Welch’s correction where appropriate. *P < 0.05, **P < 0.01 and ***P < 0.001. n.s., non-significant.

We had previously reported that ECTV lacking the viral 2′3′-cGAMP nuclease vSlfn (ECTV.ΔvSlfn) is severely attenuated and unable to prevent STING and IRF3 activation in response to DNA or 2′3′-cGAMP [12]. To assess the role of poxin on IRF3 activation during VACV infection, we engineered a recombinant VACV lacking *B2R* (VACV.ΔB2). Genomic regions flanking the VACV *B2R* locus were cloned in tandem and inserted into VACV WR in lieu of the natural *B2R* gene by transient dominant selection. Genetic analysis using primers on the flanking regions confirmed the loss of the *B2R* gene in the resulting recombinant virus, yielding a product of the same size as the donor plasmid (Fig. 2a). We also generated serum against the B2 protein (see Methods). This serum was immunoreacted with a protein of the expected size in VACV-infected, but not in VACV.ΔB2-infected, lysates (Fig. 2b). The engineered VACV.ΔB2 showed no defect in replication (Fig. 2c) or spread (Fig. 2d) in BS-C-40 cells. When infecting THP-1-IFIT-1-Gluc cells, no activation of the IFIT-1-Gluc reporter was observed upon either parental or VACV.ΔB2 infection, although a small but reproducible difference between the viruses was observed (Fig. 2e). However, when infected cells were challenged with exogenous HT-DNA or 2′3′-cGAMP, VACV.ΔB2 was impaired in its ability to suppress the IFN response (Fig. 2e). This was in line with the data reported by Eaglesham *et al*. where VACV carrying GFP in lieu of *B2R* was unable to degrade 2′3′-cGAMP [9].

**Fig. 3.**
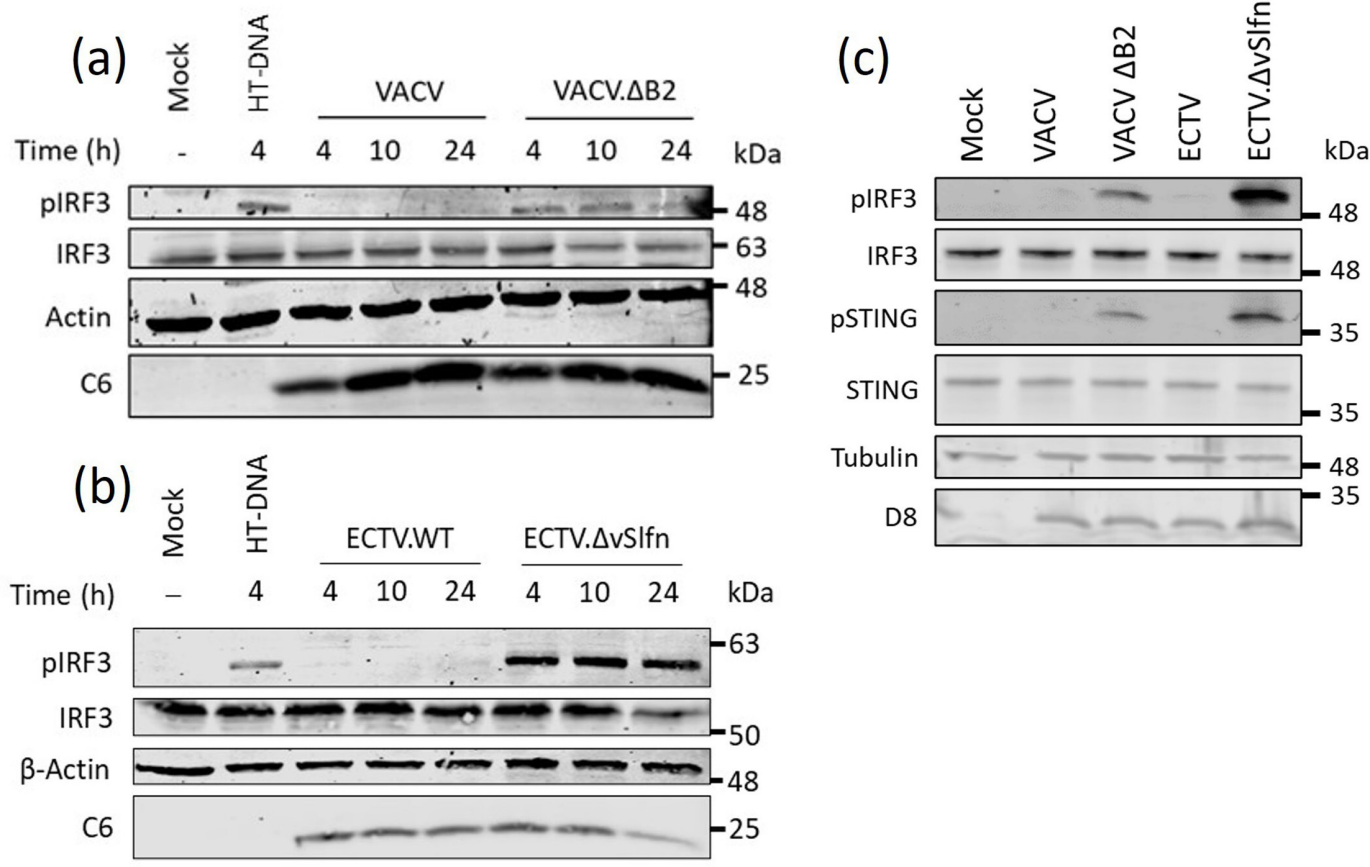
VACV and ECTV lacking poxin induce differential IRF3 activation. PMA-differentiated THP-1 cells were infected with (a) WT VACV or VACV.ΔB2, or (b) WT ECTV or ECTV.ΔvSlfn at MOI 10 for 4, 10 and 24 h. The cells were lysed with RIPA buffer and subjected to immunoblotting against phosphorylated and total levels of IRF3, β-actin and the viral protein C6. (c) Side-by-side infection of the indicated viruses (MOI 10 for 10 h) in differentiated THP-1 cells. Lysates were probed against phosphorylated and total levels of IRF3 and STING, as well as β-actin and the viral protein D8. Data are representative of at least two experiments. Molecular weight markers are shown.

We then assessed the ability of VACVΔB2 to induce the activation of STING and IRF3 by phosphoblotting. Differentiated THP-1 cells were infected with WT VACV or VACV.ΔB2 at multiplicities of infection (MOIs) 10 for 4, 10 and 24 h. Infection with VACV.ΔB2 resulted in elevated levels of pIRF3 as early as 4 h, and these were not observed with WT VACV (Fig. 3a). None of the viruses affected the total levels of IRF3, which remained constant. Immunoblotting for early protein C6 confirmed equivalent levels of infection between the WT and deletion viruses. The transfection of HT-DNA, a potent agonist of cGAS, was used as a positive control, and, as expected, it resulted in elevated levels of pIRF3. We also performed an identical experiment assessing the levels of pIRF3 during ECTV and ECTV.ΔvSlfn infections. As observed previously [12], ECTV.ΔvSlfn induced detectable levels of pIRF3 compared to WT ECTV (Fig. 3b). We noticed, however, that these were comparatively higher than those observed during VACV.ΔB2 infection. This observation was confirmed when parallel infections were conducted: ECTV.ΔvSlfn infection resulted in significantly higher pSTING and pIRF3 than VACV.ΔB2 infection in conditions in which the levels of infection were identical as revealed by D8 immunoblotting (Fig. 3c). Taken together, these results demonstrated that OPXV lacking the cGAMP nuclease orthologue gene activates the cGAS/STING pathway, although not to the same extent, hence indicating the existence of extragenic mechanisms modulating STING activation.

### Blocking genome replication reduces IRF3 activation induced by poxin-deficient viruses

**Fig. 4.**
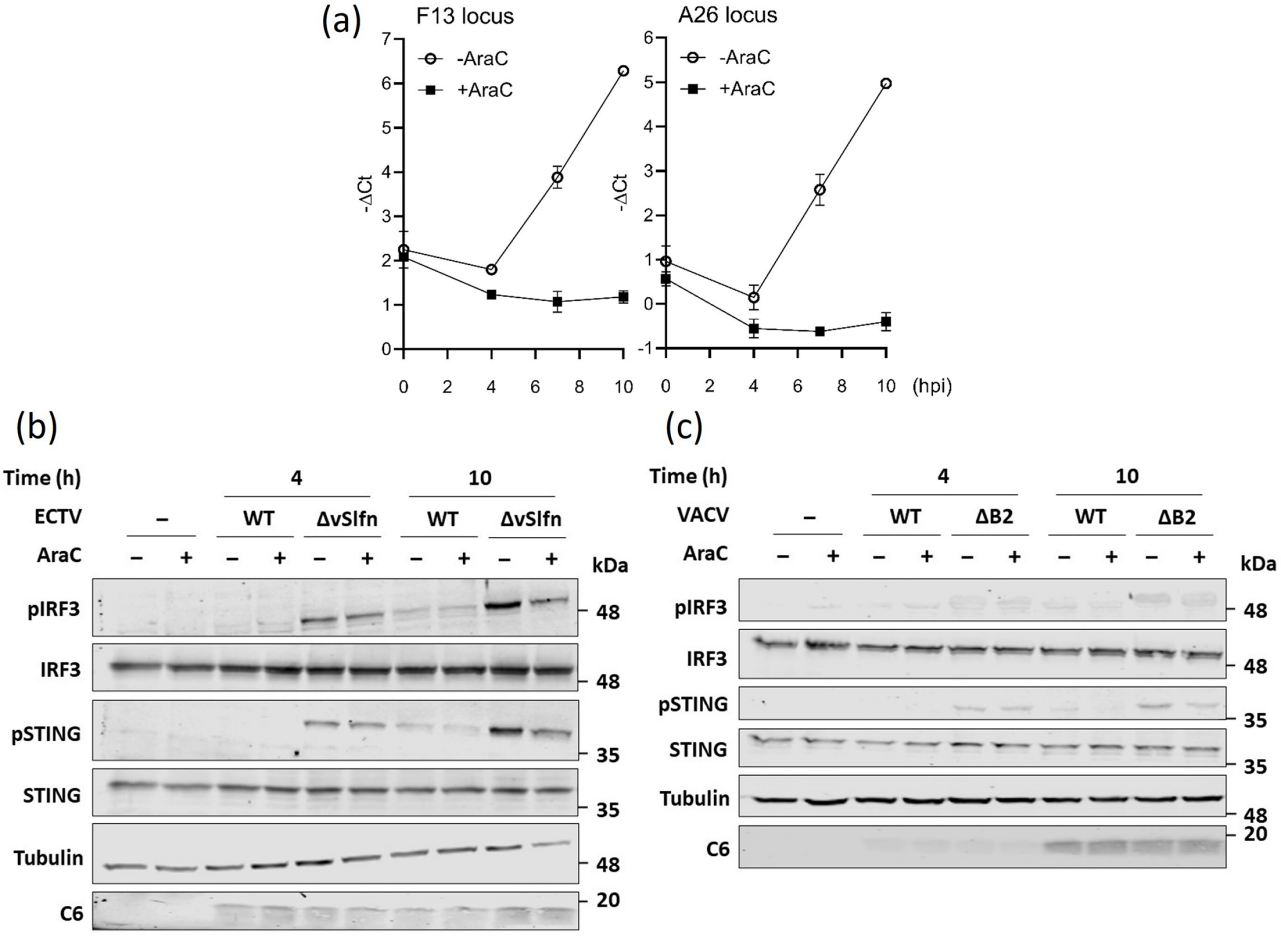
Blocking genome replication reduces IRF3 activation induced by poxin-deficient viruses. (a) Differentiated THP-1 cells were infected with 10 p.f.u. cell^−1^ of VACV in the presence or absence of 40 µg ml^−1^ AraC, and viral genomic DNA was measured at the indicated hpi by qPCR on the *F13* and *A26* loci. Differentiated THP1 cells were infected with 10 p.f.u. cell^−1^ of (b) WT ECTV or ECTV.ΔvSlfn or (c) WT VACV or VACV.ΔB2 in the presence or absence of 40 µg ml^−1^ AraC and harvested at 4 and 10 hpi. The cells were lysed with RIPA buffer and subjected to immunoblotting against the indicated proteins. Data are representative of at least two experiments. Molecular weight markers are shown.

Most work on OPXV and cytosolic DNA sensing has used replication-defective MVA because OPXV blocks this pathway with poxin, whereas MVA is naturally deficient for this protein [9,51]. Poxin-deficient OPXV such as VACV or ECTV, are therefore useful tools to understand DNA sensing of replication-competent OPXV in relevant cell types. A strategy to elucidate how OPXV lacking poxin triggers innate immune activation is to use pharmacological inhibitors that block the virus life cycle at a particular stage. Poxviruses produce large amounts of DNA in the cell cytosol during the replication of their large DNA genomes. To establish whether IRF3 activation during poxin-deficient OPXV infection derived from the recognition of replicated genomes, we used the DNA synthesis inhibitor AraC. The differentiated THP-1 cells were infected with 10 p.f.u. cell^−1^ of ECTV.ΔvSlfn or ECTV.WT in the presence or absence of 40 µg ml^−1^ of AraC and harvested at time intervals for immunoblotting. This dose of AraC has been confirmed to block genome replication (Fig. 4a). At 4 hpi, ECTV.ΔvSlfn triggered IRF3 and STING activation, and this was not reduced in the presence of AraC, indicating that DNA sensing occurred prior to genome replication (Fig. 4b). At 10 hpi, activation of STING and IRF3 was stronger, and this was reduced in the presence of AraC (Fig. 4b). Importantly, AraC did not affect the levels of *α*-tubulin or the viral early protein C6, which was used as a marker for infection. When similar experiments were performed with VACV, an overall diminished activation of STING and IRF3 was observed upon infection of VACV.ΔB2 in line with previous data comparing VACV and ECTV. Despite this reduced induction, similar results were observed in which AraC reduced activation at 10 hpi (Fig. 4c). Overall, this indicated that replicated genomes are sensed by cGAS in the absence of poxin, but some are sensed prior to replication in THP-1 cells.

### Blocking genome replication completely abolishes IRF3 activation in human fibroblasts

**Fig. 5.**
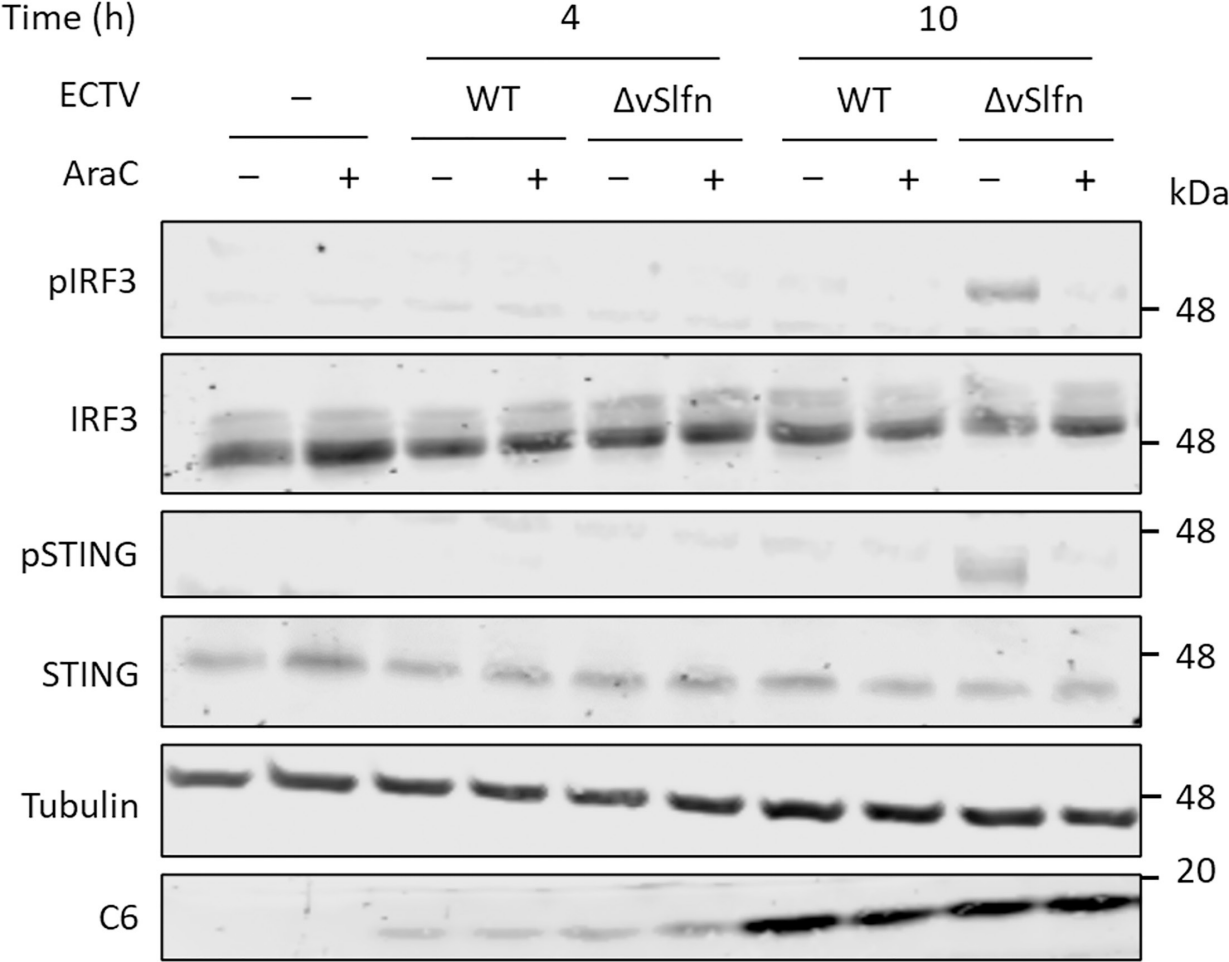
Blocking genome replication completely abolishes IRF3 activation in human fibroblasts. Immortalized human primary fibroblasts were infected with WT ECTV or ECTV.ΔvSlfn in the presence or absence of 40 µg ml^−1^ of AraC and harvested at 4 and 10 hpi. The cells were lysed with RIPA buffer and subjected to immunoblotting against the indicated proteins. Data are representative of at least three experiments. Molecular weight markers are shown.

To further investigate the role of genome replication in the sensing of poxviruses, experiments were repeated in a second cell type. We took human primary fibroblasts (HFFF) that have been immortalized with human telomerase reverse transcriptase (HFFF-nTERT) as a relevant cell type for skin-tropic viruses, such as OPXV. As observed in THP-1 cells, ECTV.WT infection resulted in no detectable levels of pSTING or pIRF3 (Fig. 5). Infection with ECTV.ΔvSlfn, however, resulted in STING and IRF3 activation at 10 hpi, and this was abolished by AraC (Fig. 5). This indicated that replicated genomes contribute to OPXV immune sensing in human fibroblasts. Interestingly, and in contrast to THP-1 cells, neither pIRF3 nor pSTING was detected at 4 hpi in infected HFFF-nTERT cells. This suggested that macrophages, but not fibroblasts, are able to sense the incoming virions prior to genome replication.

### Uncoated genomes contribute to IRF3 activation in differentiated THP-1 cells

Upon entry, the OPXV genome is released from the incoming virions and deposited in the cytosol in a process that requires the expression and translation of the viral uncoating factor D5 [14,21]. Cycloheximide (CHX) is a translation inhibitor that functions by interfering with the translocation of tRNA molecules and mRNA in relation to the ribosome so elongation cannot take place, thus blocking new protein synthesis. We initially explored the use of CHX to prevent genome uncoating in macrophages and demonstrated the sensing of incoming released genomes in these cells. However, CHX treatment resulted in enhanced IRF3 activation in the absence of viral infection upon DNA sensing stimulation for reasons that remain unclear (not shown). We, therefore, applied a genetic approach knocking down the expression of *D5L* via RNAi. An shRNA sequence against D5, the viral uncoating factor, and an shControl sequence (shCtrl) were designed and cloned into a lentivector encoding puromycin resistance. Lentiviral particles were produced in HEK293T cells and used to transduce THP-1 cells. Following lentivirus transduction, these cells were clonally selected in the presence of puromycin.

**Fig. 6.**
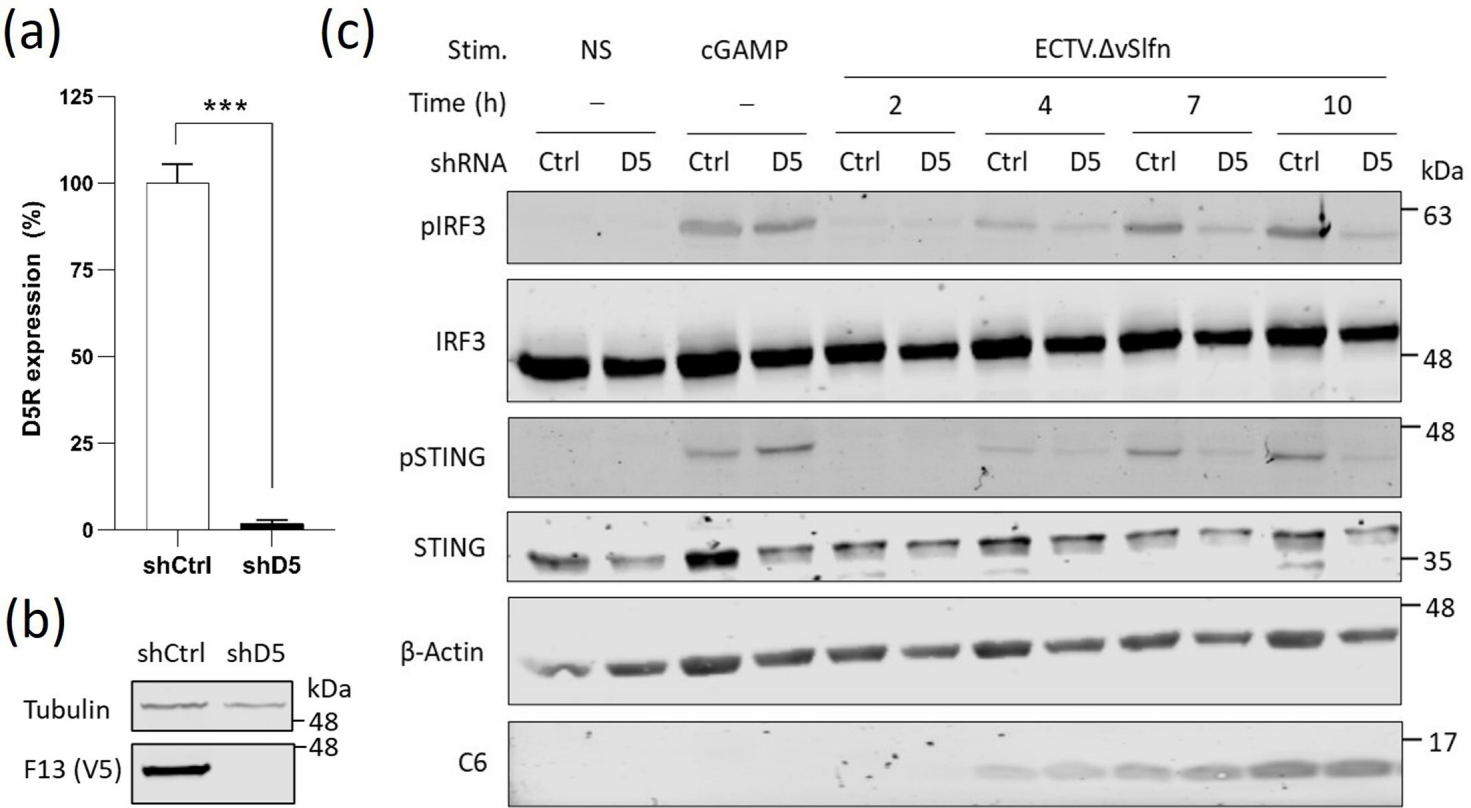
Uncoated genomes contribute to IRF3 activation in differentiated THP-1 cells. (a) THP-1 cells were transduced with lentiviruses carrying an shRNA against the viral gene *D5R* (shD5) or a control sequence (shCtrl). Abundance of D5R mRNA upon infection was measured by qPCR and presented as a percentage over shCtrl cells. (b) shCtrl and shD5 cells were infected with a recombinant VACV carrying V5-tagged F13 [35] and the lysates were subjected to immunoblotting against V5 and α-tubulin. (c) shCtrl and shD5 cells were infected with WT ECTV or ECTV.ΔvSlfn for the indicated lengths of time. Non-stimulated (NS) cells and cells transfected with cGAMP were used as controls. The cells were lysed with RIPA buffer and subjected to immunoblotting against the indicated proteins. Data are representative of three experiments. Molecular weight markers are shown. NS, non-simulated. ***P < 0.001.

To assess the level of expression of D5, shD5 and shCtrl, cells were differentiated for 48 h and infected with VACV for 24 h. The cells were then harvested for RNA extraction, followed by cDNA synthesis and qPCR using 18S RNA and D5 primers. The -ΔCt value was calculated and presented as a percentage of D5 expression in mock-infected cells (Fig. 6a). This showed an almost complete knock-down, with a residual 2% D5 expression in shD5 cells. Similarly, shD5 and shCtrl cells were infected with a recombinant VACV expressing V5-tagged F13 [35], an intermediate gene whose expression requires genome uncoating and replication. F13 expression was shown to be abrogated in shD5 cells (Fig. 6b). We then assessed the impact of blocking uncoating on the sensing of poxvirus infection. shCtrl and shD5 were infected with ECTV.ΔvSlfn for different lengths of time. Consistent with previous results, ECTV.ΔvSlfn infection resulted in detectable levels of pSTING and pIRF3 at all time points tested, with robust activation at 4 hpi. However, and in contrast with previous data, where AraC treatment did not affect IRF3 activation at 4 hpi, D5 depletion resulted in substantially lower levels of pIRF3 and pSTING (Fig. 6d). As a control, IRF3 and STING activation was equivalent in shCtrl and shD5 cells in response to 2′3′-cGAMP. These results demonstrated that viral genomes are exposed to cellular sensors upon uncoating and can be sensed prior to genome replication.

## Discussion

Viruses require *de novo* synthesis of viral proteins to modify the host environment and ensure effective takeover for replication. A prerequisite to forming replication factories is the release of the genome from the incoming virion, a stage of the virus life cycle particularly vulnerable to immune sensors and nucleases. To counteract this, many, if not all, viruses package key host-response modifiers and antagonists into viral particles so they are immediately deployed upon entry. In addition, viruses go to great lengths to hide and protect their genome within the intracellular environment. For instance, the human immunodeficiency virus (HIV) recruits cellular cofactors and metabolites to the capsid to cloak newly synthesized viral DNA from cGAS recognition during transit to the nucleus, where genome integration occurs [42,52, 53]. Equally, flavivirus replication complexes located within the vesicle pockets from the ER limit the exposure to RIG-I-like receptor recognition [54,55]. In the case of poxviruses, it is well-established that the viral lateral bodies carry host-response modifiers including the immunomodulatory H1 phosphatase and that these are released upon core disassembly [56,59]. It is also established that poxvirus replication occurs in close association with ER membranes, likely protecting the parental genome [29,30]. Data presented here show that the poxvirus uncoating process operates in a similar manner, hiding the genome from cGAS while the early gene expression takes place. Our results show that the deletion of the single gene encoding the viral cGAMP nuclease or poxin results in a potent activation of STING and IRF3, and that this is induced by both incoming and replicated viral genomes. Given that poxin acts by degrading 2′3′-cGAMP, it implies that the viral genome is effectively sensed by cGAS pre- and post-replications. *Poxin* is an early gene, and there is no evidence that it is associated with the lateral bodies [58,62]. Poxvirus early genes are transcribed from within the core before genome release takes place [16,20]. Early genes not only encode the proteins needed for the subsequent phase of gene expression and replication but also the viral inhibitors needed to suppress cellular responses to infection, such as poxin. Given that the uncoating process is timed to liberate the genome only when the large array of viral early genes has been expressed, the viral core effectively acts as an immune evasion strategy, hiding the genome until viral host modifiers are deployed.

In addition to poxin, a large array of early genes with immunomodulatory properties is expressed during poxvirus infection, many of which prevent activation of NF-κB and IFN pathways [7,63]. The strong activation of IRF3 and attenuation in virulence observed by single-gene deletion of poxin highlights the importance of this gene in poxvirus immune evasion [12]. Indeed, poxin is conserved in all OPXV as a single gene or fused to a Slfn domain, with the exception of variola virus (VARV), where the whole ORF is inactivated [9,51]. Counterintuitively, although poxin homologs can be found in the genomes of other large DNA viruses, such as baculoviruses, many vertebrate poxviruses, including parapoxviruses, leporipoxviruses, capripoxviruses or avipoxviruses lack this gene [9,51]. It remains unclear how poxvirus species in these genera deal with cGAS-STING activation in the absence of poxin and whether they have evolved alternative strategies to target this pathway, although a recent report uncovered the existence of phage-like phosphodiesterases in avipoxviruses able to suppress 2′3′-cGAMP IFN activation [64]. Interestingly, our results demonstrate that in the absence of poxin, ECTV infection results in stronger IRF3 activation than VACV, revealing the existence of extragenic mechanisms to contend with cGAS/STING activation. In addition to poxin, the viral proteins F17 and E5 have also been shown to target this pathway. Protein F17 is a late protein packaged into virions that profoundly dysregulates cGAS sensing by targeting the mammalian target of rapamycin [49,65]. Protein E5 is an early protein necessary for cGAS degradation [66]. F17 is highly conserved across OPXV species and poxvirus genera, only absent in simpler species, such as salmon gill and crocodile poxviruses, whereas E5 is far less conserved and only present in leporipoxviruses, centapoxviruses and OPXV, with the notable exception of MPXV [67]. Given the presence of both F17 and E5 in ECTV and VACV, it is likely that other genes account for the differential STING activation observed between these viral species in the absence of poxin.

Our results also highlight differences in the sensing between fibroblasts and macrophages. Whereas STING activation in fibroblasts occurred at 10 hpi and was completely abolished by AraC treatment, in macrophages, the sensing occurred much earlier and was only partially sensitive to AraC (Figs4  5). This indicated that the macrophages were equipped to sense incoming released viral genomes, which we confirmed by blocking genome release via depletion of D5 expression. These observations are likely to be explained by qualitative and quantitative differences in the expression of cGAS, co-factors operating with cGAS and the repertoire of restriction factors that are expressed by macrophages relative to fibroblasts. Interestingly, infection of macrophages resulted in low but reproducible STING and IRF3 activation even when D5 expression was abolished (Fig. 6). At present, the PAMP resulting in STING and IRF3 activation in this context remains unclear. Treatment of the purified virus stocks with benzonase (a promiscuous endonuclease that degrades all forms of DNA and RNA including single-stranded, double-stranded, linear and circular forms) did not alter IRF3 activation (data not shown), indicating that no nucleic acids were co-purified with viral particles. It is possible that an endogenous PAMP was released in response to the infection-mediated STING activation. For instance, it has been reported that stress can induce changes in the morphology of mitochondria and multiple viruses can cause leakage of mitochondrial DNA into the cytosol resulting in cGAS activation and induction of innate responses [68,71]. OPXV infection, however, sustains mitochondrial activity and does not induce mitochondrial apoptosis [72], and a recent study has reported the absence of mitochondrial DNA release in cells infected with VACV, at least during the initial 6 h of infection [73]. Alternatively, the source of DNA triggering cGAS activation could derive from the premature or non-canonical uncoating of viral particles. This premature uncoating could represent naturally occurring defective particles or could be induced by host factors. Interestingly, TRIM5α (tripartite motif-containing protein 5 alpha, a restriction factor that binds the incoming retroviral capsid driving the premature uncoating of HIV-1) has recently been shown to restrict OPXV via a mechanism that requires binding to the OPXV capsid protein L3 [74]. Whether TRIM5α synergizes with cGAS to restrict virus infection and induce innate activation remains to be tested.

In summary, this report shows that the infection with two different OPXV species (ECTV and VACV) has the capacity to induce STING and IRF3 activation in at least two different cell types and that this is mediated by released as well as replicated viral genomes. STING and IRF3 activation are, however, prevented by the viral cGAMP nuclease, demonstrating that the viral core and the process of uncoating are critical in protecting the viral genome from cellular recognition while early gene expression takes place. The study also reveals a third mechanism to activate cGAS that occurs before viral genome release. Although this study does not enable the definition of this agonist, our observations indicate that cGAS is activated by multiple means during OPXV infection, explaining the critical role of viral antagonists of the cGAS–STING pathway in virus virulence.

## Abbreviations

AraC: cytosine arabinoside
2′3′-cGAMP: cyclic 2′3′-GMP-AMP
cGAS: cyclic 2′3′-GMP-AMP (2′3′-cGAMP) synthase
CHX: cycloheximide
COP: copenhagen
CPXV: cowpox virus
DMEM: Dulbecco’s modified eagle medium
ECTV: *Ectromelia* virus
ER: endoplasmic reticulum
GLuc: *Gaussia* luciferase
HFFF: human foetal foreskin fibroblasts
HIV: human immunodeficiency virus
hpi: hours post-infection
HT-DNA: Herring Testes DNA
IFIT1: IFN-induced protein with tetratricopeptides 1
IFN: interferon
IRF: IFN responsive factor
LPS: lipopolysaccharide
MAVS: mitochondrial antiviral signalling
MOI: multiplicities of infection
MPXV: monkeypox virus
MVA: modified vaccinia Ankara
OPXV: orthopoxvirus
PAMP: pathogen-associated molecular pattern
pIRF3: phosphorylated IRF-3
PMA: phorbol 12-myristate 13-acetate
poxin: poxvirus immune nuclease
PRR: pattern-recognition receptors
pSTING: phosphorylated STING
pTBK1: phosphorylated TBK1
RIG-1: retinoic acid-inducible gene
RIPA: radioimmunoprecipitation assay
RPMI: Roswell Park Memorial Institute
SeV: Sendai virus
shCtrl: shControl sequence
shRNA: short hairpin RNA
STING: stimulator of IFN genes
TBK−1: TANK-binding kinase-1
TRIM5α: tripartite motif-containing protein 5 alpha
VACV: vaccinia virus
vSlfn: viral Schlafen
WR: Western Reserve
